# Non-Volatile Component and Antioxidant Activity: A Comparative Analysis between *Litsea cubeba* Branches and Leaves

**DOI:** 10.3390/molecules29040788

**Published:** 2024-02-08

**Authors:** Wei Dai, Boyi Li, Yanli Xiong, Liping Dai, Yuan Tian, Liangqian Zhang, Qi Wang, Guoqiang Qian

**Affiliations:** 1 Teaching and Experimental Center, Guangdong Pharmaceutical University, Guangzhou 510006, China; dai_gdpu_2018@gdpu.edu.cn (W.D.);; 2 School of Chinese Medicine, Guangdong Pharmaceutical University, Guangzhou 510006, Chinaxiongyanli106@163.com (Y.X.); 3 Key Laboratory of Xinjiang Phytomedicine Resource and Utilization, Ministry of Education, School of Pharmacy, Shihezi University, Shihezi 832003, China

**Keywords:** *Litsea cubeba*, non-volatile component, antioxidant activity, UPLC-HRMS

## Abstract

*Litsea cubeba*, which is found widely distributed across the Asian region, functions as both an economic tree and a medicinal plant with a rich historical background. Previous investigations into its chemical composition and biological activity have predominantly centered on volatile components, leaving the study of non-volatile components relatively unexplored. In this study, we employed UPLC-HRMS technology to analyze the non-volatile components of *L. cubeba* branches and leaves, which successfully resulted in identifying 72 constituents. Comparative analysis between branches and leaves unveiled alkaloids, organic acids, and flavonoids as the major components. However, noteworthy differences in the distribution of these components between branches and leaves were observed, with only eight shared constituents, indicating substantial chemical variations in different parts of *L. cubeba*. Particularly, 24 compounds were identified for the first time from this plant. The assessment of antioxidant activity using four methods (ABTS, DPPH, FRAP, and CUPRAC) demonstrated remarkable antioxidant capabilities in both branches and leaves, with slightly higher efficacy observed in branches. This suggests that *L. cubeba* may act as a potential natural antioxidant with applications in health and therapeutic interventions. In conclusion, the chemical composition and antioxidant activity of *L. cubeba* provides a scientific foundation for its development and utilization in medicine and health products, offering promising avenues for the rational exploitation of *L. cubeba* resources in the future.

## 1. Introduction

*Litsea cubeba*, a member of the Lauraceae family in the *Litsea* genus, thrives primarily in China, India, Thailand, and various Asian countries [1]. Despite occupying a mere 0.04% of China’s wild forest area, the *L. cubeba* tree stands out for its rich plant resources [2]. Recognized for its economic significance, *L. cubeba* has been an integral part of traditional Chinese folk medicine for generations. Its roots, stems, leaves, flowers, and fruits have been employed for medicinal purposes, offering remedies for dispelling wind and cold, reducing swelling, and alleviating pain [1,3].

A considerable amount of research has delved into unraveling the chemical constituents and biological activities of *L. cubeba*, with a predominant focus on its volatile components, especially the essential oil. The fruit and leaves of *L. cubeba*, rich in essential oils, exhibit a diverse composition of monoterpenes and sesquiterpenes [4]. Notably, citral predominates, widely used in toothpaste, soap, perfumes, and various pharmaceutical and chemical products [5,6,7]. While volatile components have received significant attention, there remains a noteworthy gap in exploring non-volatile components. Over 150 non-volatile components, including alkaloids, lignans, flavonoids, and terpenoids, have been identified in *L. cubeba*. Alkaloids and lignans, constituting over half of these components, emerge as prominent contributors with significant pharmacological activities [8,9,10,11]. Aporphine alkaloids, in particular, have demonstrated hypoglycemic, anti-inflammatory, and anti-cancer effects [12]. Despite these promising findings, research on non-volatile components remains comparatively limited, overshadowed by the predominant focus on essential oils. The imperative for an in-depth exploration of *L. cubeba*’s active ingredients and their potential pharmacological effects is evident.

Oxidative stress, implicated in the onset and progression of numerous diseases, poses a significant threat to human health, particularly in the context of neurodegenerative and cardiovascular diseases [13]. Addressing this challenge involves exploring exogenous antioxidants or enhancing endogenous antioxidant defenses. Plants, recognized as valuable repositories of secondary metabolites, emerge as promising sources of natural antioxidants. Studies underscore the potential of *L. cubeba* as a natural antioxidant, with its essential oil showcasing robust antioxidant activity [14,15]. Extracts from *L. cubeba* bark, including methanol, chloroform, *n*-butanol, and water extracts, also exhibit antioxidant activity [16].

Given the abundant resources of *L. cubeba*, and the relatively limited research on its non-volatile components, there is a need for a more comprehensive exploration to facilitate the rational development and utilization of this plant resource. This is particularly crucial for uncovering its broader medicinal value, with a specific focus on discovering its potential as a robust antioxidant. This study endeavors to bridge this knowledge gap, focusing on the branches and leaves of *L. cubeba*. Employing UPLC-HRMS, we aim to comprehensively identify non-volatile components in the methanol extract. The antioxidant activity will be assessed through standardized assays, including ABTS (2,2′-azino-bis(3-ethylbenzothiazoline-6-sulphonic acid)), DPPH (1,1-diphenyl-2-trinitrophenylhydrazine), FRAP (ferric reducing antioxidant power), and CUPRAC (cupric reducing antioxidant capacity). By meticulously comparing and analyzing differences in non-volatile components and antioxidant activity, this study aspires to provide a fresh perspective for the rational development and utilization of *L. cubeba* resources, potentially unlocking novel avenues in medicinal research.

## 2. Results

### 2.1. UPLC-HRMS Analysis of Non-Volatile Components from L. cubeba Branches and Leaves

The chemical composition of the methanol extracts from *L. cubeba* branches and leaves was analyzed using UPLC-Q-Orbitrap HRMS in both positive and negative ion modes. Figures S1–S4 in the Supplementary Materials present the total ion chromatograms of mass spectrometry analysis for the branches and leaves of *L. cubeba*. Detailed identification information for the compounds is provided in Table 1. A total of 72 compounds were identified in *L. cubeba*, comprising 23 alkaloids, 15 flavonoids, 14 organic acids, five amino acids, four alcohols, three carbohydrates, two ketones, two phenols, two esters, one ether, and one phenylpropanoid. Alkaloids, flavonoids, and organic acids emerged as the major chemical constituents of *L. cubeba*. Specifically, 36 and 44 compounds were identified in the branches and leaves of *L. cubeba*, respectively, with 24 compounds being identified for the first time from this plant. The discovery of these compounds significantly contributes to the chemical information available for *L. cubeba*.

#### 2.1.1. Analysis of Non-Volatile Components in Branches

Within the *L. cubeba* branches, a total of 36 non-volatile compounds were identified, encompassing 16 alkaloids (No. 15, 23, 24, 26, 27, 28, 30, 32, 33, 35, 39, 41, 53, 54, 58, and 59). These alkaloids comprised 11 isoquinoline alkaloids (No. 15, 23, 24, 28, 30, 32, 33, 35, 39, 41, and 53), two indole alkaloids (No. 26 and 27), two amide alkaloids (No. 54 and 59), and one other alkaloid (No. 59). Additionally, seven flavonoids (No. 36, 37, 44, 46, 47, 48, and 49), five organic acids (No. 56, 65, 66, 67, and 68), four alcohols (No. 60, 64, 69, and 70), two esters (No. 61 and 62), one ether (No. 14), and one phenylpropanoid (No. 52) were identified. Alkaloids constituted the major component, accounting for nearly 45% of the identified compounds. The sum of flavonoids, organic acids, and alcohol compounds also represented approximately 45% of the total identified compounds in the branches.

#### 2.1.2. Analysis of Non-Volatile Components in Leaves

In *L. cubeba* leaves, a total of 44 non-volatile compounds were identified, including 12 alkaloids (No. 1, 12, 17, 18, 30, 32, 33, 45, 54, 58, 63, and 72). Among the alkaloids, four were isoquinoline alkaloids (No. 30, 32, 33, and 45), two were amide alkaloids (No. 1 and 54), two were pyridine alkaloids (No. 17 and 18), one was a purine alkaloid (No. 12), one was a pyrrolidine alkaloid (No. 72), one was a quinoline alkaloid (63), and one was classified as other (No. 58). Additionally, 10 organic acids (No. 3, 5, 8, 9, 16, 19, 20, 25, 31, and 56), nine flavonoids (No. 34, 36, 38, 42, 43, 50, 51, 55, and 57), five amino acid compounds (No. 6, 10, 11, 21, and 22), three carbohydrates (No. 2, 4, and 7), two phenols (No. 29 and 40), two ketones (No. 29 and 40), and one alcohol were identified. Alkaloids, organic acids, and flavonoids were the major constituents of *L. cubeba* leaves, constituting over 70% of the total identified compounds.

### 2.2. Comparative Analysis of Non-Volatile Components in Branches and Leaves

**Figure 1:**
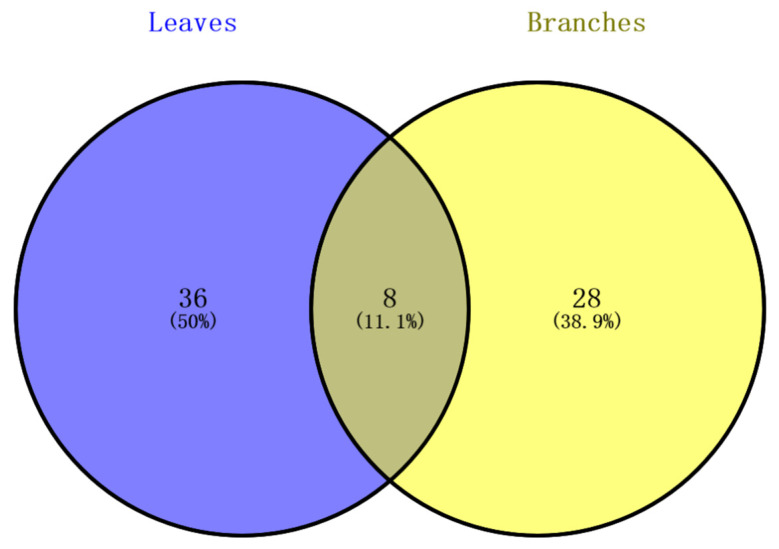
Venn diagram of the non-volatile components of *L. cubeba* branches and leaves.

The comparative analysis of non-volatile components in the methanol extracts of *L. cubeba* branches and leaves revealed substantial differences in their chemical compositions (Figure 1). Out of the 72 identified compounds in *L. cubeba*, only eight (No. 30, 32, 33, 36, 54, 56, 58, and 64) were common to both branches and leaves. Leaves displayed 36 unique compounds (No. 1, 2, 3, 4, 5, 6, 7, 8, 9, 10, 11, 12, 13, 16, 17, 18, 19, 20, 21, 22, 25, 29, 31, 34, 38, 40, 42, 43, 45, 50, 51, 55, 57, 63, 71, and 72), while branches had 28 unique compounds (No. 14, 15, 23, 24, 26, 27, 28, 35, 37, 39, 41, 44, 46, 47, 48, 49, 52, 53, 59, 60, 61, 62, 65, 66, 67, 68, 69, and 70). These distinctive compounds underscore pronounced differences in their chemical profiles.

Alkaloids constituted a major component in both branches and leaves, sharing several compounds such as No. 30, 32, 33, 54, and 58. However, variations exist in the types of alkaloids, with branches primarily featuring isoquinoline alkaloids and leaves encompassing isoquinoline, amide, and other alkaloid categories. Organic acids were prevalent in both branches and leaves, with compound 56 being the sole shared compound (15-Hexadecynoic acid). Nevertheless, other organic acid components exhibited significant differences. Flavonoids, another major class, were identified in both parts, with only Rutin (compound 36) being a shared compound. Other flavonoids were unique to either branches or leaves. Notable distinctions were observed in alcohol and ester content, with branches featuring a higher representation of compounds (No. 60, 64, 69, 70, 61, and 62). Amino acids were exclusively found in leaves (No. 6, 10, 11, 21, and 22). Phenols and ketones were unique to leaves (No. 29 and 40).

In conclusion, while alkaloids, organic acids, and flavonoids were major components in both branches and leaves, the number of shared components was limited. The presence of unique compounds in each part contributed significantly to their distinct chemical profiles, enhancing our understanding of the chemical diversity of *L. cubeba* and the potential biological activities associated with its various components.

### 2.3. Antioxidant Activity

#### Results of Four In Vitro Antioxidant Activity Tests

The evaluation of in vitro antioxidant activity involves various methods based on different principles, with the most commonly utilized ones being ABTS, DPPH, FRAP, and CUPRAC. ABTS and DPPH assess the antioxidant capacity of samples through measuring absorbance change resulting from the reduction of their respective free radicals under the influence of oxidants. In contrast, FRAP and CUPRAC characterize the activity of antioxidants through reducing metal ions to lower oxidation states and measuring their generation. The comprehensive application of these methods provides a nuanced understanding of antioxidant potential within samples. In this study, we assessed the in vitro antioxidant activity of methanol extracts from both branches and leaves of *L. cubeba* using ABTS, DPPH, FRAP, and CUPRAC methods. For the ABTS, DPPH, and FRAP experiments, antioxidant capacity was expressed as TEAC µmol/g, where higher values signify greater antioxidant capability. For the FRAP method, results were indicated as Fe (Ⅱ) µmol/g, with higher values reflecting stronger reducing power and thus greater antioxidant potential. The methanol extracts from *L. cubeba* branches exhibited the following results for the four antioxidant assays, as shown in Table 2. These antioxidant activity data indicate that both branches and leaves of *L. cubeba* possess considerable antioxidant capabilities, with a slight predominance in the antioxidant capacity of the branches over the leaves.

## 3. Discussion

In this investigation, UPLC-HRMS technology facilitated the rapid and precise analysis of non-volatile components within *L. cubeba* branches and leaves. The established analytical method for non-volatile components offered insights into the diverse compounds present in these plant parts, contributing detailed information to enhance our understanding of the plant’s chemical characteristics. The identification of 72 compounds, including alkaloids, flavonoids, and organic acids, underscored the chemical diversity of *L. cubeba*. Moreover, methanol extracts from both branches and leaves exhibited noteworthy antioxidant activity in vitro.

While the results align with some previously reported chemical compositions, this study uncovered 24 compounds not documented before, enriching our comprehensive understanding of *L. cubeba* compounds and supporting its chemical taxonomy, development, and utilization [74]. Alkaloids, especially isoquinoline alkaloids, emerged as significant components in the non-volatile fraction of *L. cubeba*, aligning with prior research [74]. Intriguingly, a higher abundance of isoquinoline alkaloids in branches compared to leaves was observed. Further analysis of alkaloid differences identified eight shared compounds, five of which belonged to the alkaloid category (compounds 30, 32, 33, 54, and 58). The predominance of isoquinoline alkaloids in branches, and the greater diversity of alkaloid types in leaves, suggest potential variations in pharmacological activities. Known for their diverse biological activities, especially in pharmaceutical research, the abundance of isoquinoline alkaloids in *L. cubeba* implies their essential role in its medicinal effects.

Flavonoids, renowned for their antioxidant properties, were identified as major components in both branches and leaves of *L. cubeba*. The presence of the shared compound rutin (compound 36) suggests common antioxidant capabilities. However, unique flavonoid compositions in each part may contribute to subtle differences in antioxidant activity. The study also confirmed the presence of organic acids, a less explored aspect of *L. cubeba* chemistry. The only shared organic acid between the branches and leaves was 15-hexadecynoic acid (compound 56), indicating potential variations in their health-promoting properties. Beyond the primary compound categories, differences in alcohols, esters, amino acids, and phenols between the branches and leaves add layers to the plant’s chemical diversity, potentially influencing related biological activities and the potential medicinal value of *L. cubeba*.

The assessment of antioxidant potential using ABTS, DPPH, FRAP, and CUPRAC assays demonstrated significant antioxidant capabilities in both the branch and leaf extracts, with a slightly higher antioxidant capacity observed in the branches. In comparison with antioxidant capacities of traditional medicinal plants, such as *Angelica dahurica*, *Fritillaria cirrhosa,* and *Perilla frutescens*, *L. cubeba* branches and leaves exhibited superior or comparable antioxidant abilities [75]. The plant’s rich content of alkaloids, flavonoids, and organic acids, known for their antioxidant properties, likely contributes to its robust antioxidant activity, emphasizing the potential health benefits of *L. cubeba* in traditional herbal applications.

In summary, this comparative analysis highlights the diverse chemical characteristics and antioxidant capabilities of *L. cubeba* branches and leaves. As a botanical resource, *L. cubeba* exhibits unique features in terms of chemical composition and antioxidant activity, providing a theoretical foundation for its applications in medicine and the food industry. Future research could delve deeper into the biological activities and pharmacological effects of *L. cubeba* compounds to explore its potential health benefits. This study offers valuable information for the development and utilization of *L. cubeba*, laying a scientific foundation for its diverse applications.

## 4. Materials and Methods

### 4.1. Plant Material and Extraction

#### 4.1.1. Plant Material

*L. cubeba* was sourced from Yunfu, China, in April 2023, and Dr. Xinger Ye, affiliated with the College of Traditional Chinese Medicine Resources at Guangdong Pharmaceutical University, authenticated the plant materials.

#### 4.1.2. Preparation of Liquid-Sample Solution for Mass Spectrometry

The dried leaves and stems of *L. cubeba* were individually pulverized using the DFY–300C Swing Crusher from Wenling Linda Machinery Co., Ltd, Wenling, China. Approximately 1.0 g of each sample powder was precisely weighed into a 100 mL conical flask. Analytical methanol (50 mL) was added accurately, and the mixture was re-weighed. The Q-500DE ultrasound equipment (Dongguan Keqiao Ultrasonic Equipment Co., Ltd., Dongguan, China) was used for ultrasonic extraction at 50 °C with 500 W power and 40 kHz frequency for 30 min. After cooling, the sample was re-weighed, and any weight loss was compensated by adding methanol. Ten milliliters of the upper clear liquid were transferred to a centrifuge tube, and centrifuged at 2200× *g* for 15 min using a high-speed centrifuge. Two hundred microliters of the supernatant were extracted, mixed with 800 µL of chromatographic methanol, diluted to 1 mL, thoroughly mixed, and then filtered through a 0.22 µm microporous membrane. The resultant filtered solution served as the liquid–sample solution for mass spectrometry.

#### 4.1.3. Preparation of Samples for Antioxidant Activity Testing

For antioxidant activity testing, 1.0 g of *L. cubeba* leaves and branches powder was precisely weighed and combined with 40 mL of methanol. Ultrasonic extraction was conducted at 50 °C using an ultrasonic power of 500 W (frequency, 40 kHz) for 50 min, followed by filtration and rotary evaporation to obtain the respective extracts. The yield was 117.7 mg for leaf extract and 61.4 mg for branch extract. The extracts were dissolved in 15 mL of methanol. Based on preliminary experiments, the leaf methanol extract was diluted 10 times, and the branch methanol extract was diluted 5 times to produce the test solutions.

### 4.2. The Main Chemicals and Reagents

Analytical grade methanol from Da Mao Chemical Reagent Co., Ltd., Tianjin, China; chromatography-grade acetonitrile from Honeywell Trading (Shanghai) Co., Ltd., Shanghai, China; and distilled water from Watsons, Hong Kong, China. The total antioxidant capacity assay kits (ABTS, DPPH, FRAP methods) were obtained from Shanghai Macklin Biochemical Technology Co., Ltd., Shanghai, China, and the liquid sample total antioxidant capacity assay kit (Cu^2+^ method) was sourced from Beijing Applygen Technology Co., Ltd., Beijing, China.

### 4.3. UPLC-HRMS Analysis

#### 4.3.1. Instrumentation and Conditions

The analysis employed the Vanquish Flex UPLC system, coupled with the Orbitrap Exploris 120 quadrupole electrostatic field orbital well high-resolution mass spectrometer for UPLC-Q-Orbitrap HRMS, sourced from Thermo Fisher Scientific (Waltham, MA, USA). A Hypersil GOLD C_18_ column (100 mm × 2.1 mm, 1.9 μm) was used for chromatographic analysis with a flow rate of 0.3 mL·min^−1^, maintaining the column temperature at 35 °C. A 2.00 μL sample was injected into the system. The mobile phase comprised acetonitrile (A) and a 0.1% formic acid solution (B). The gradient elution profile was programmed as follows: starting at 95% B for 5 min, gradually decreasing to 80% B from 5 to 8 min, further decreasing to 75% B from 8 to 20 min, reducing to 5% B from 20 to 22 min, holding at 5% B from 22 to 22.001 min, and finally increasing to 95% B from 22.001 to 25 min.

The mass spectrometry analysis utilized positive and negative ion switching scan modes (Full scan/dd-MS^2^). H-ESI ionization was employed with an electrospray voltage of 3.5 kV (+)/2.8 kV (−). The ion transfer tube temperature was set at 325 °C, and the auxiliary gas temperature was maintained at 350 °C, RF Lens at 70%. Nitrogen served as nebulizing gas, auxiliary gas, and sheath gas. The flow rates for sheath gas, auxiliary gas, and sheath gas were 50 Arb, 8 Arb, and 1 Arb, respectively. Primary and secondary resolutions were set at 60,000 (MS) and 15,000 (MS^2^), respectively. The ion scan range (*m*/*z*) was 100–1500, and the collision energy was normalized. The gradient for collision energy was set at 20%, 40%, and 60%.

#### 4.3.2. Data Analysis and Identification of Compounds

The original data files were processed using Compound Discoverer 3.3 software provided by Thermo Fisher Scientific (Waltham, MA, USA), employing a compound identification method template. This involved peak area extraction, alignment, and matching secondary fragment spectra against the mzCloud network database and the local database mzVault. Filtering criteria included mass deviation control, optimal scores, and comparison with compound information in the compound library. Subsequent analysis of compounds included cross-referencing with literature and online databases (PubChem, CNKI, PubMed) for enhanced understanding.

### 4.4. Antioxidant Activity

#### 4.4.1. ABTS Radical Scavenging Assay

The ABTS assay followed the Total Antioxidant Capacity Assay Kit. ABTS solution was combined with an oxidizing agent, and the resulting mixture served as the working solution. After 12–16 h of dark storage at room temperature, the ABTS working solution underwent dilution with 80% ethanol, and the absorbance at 734 nm was measured using an Agilent Synergy H1 microplate reader (Agilent Technologies, Inc., VT, USA) until reaching 0.7 ± 0.05 absorbance units. Test and standard solutions were mixed with the ABTS working solution in a 96-well plate, and absorbance at 734 nm was recorded after incubation. Trolox served as the standard, and a series of Trolox standard solutions (0.15, 0.3, 0.6, 0.9, 1.2, 1.5 mM) were prepared. The total antioxidant capacity of each sample was expressed as µmol Trolox/g of herbal medicine dry weight [76].

#### 4.4.2. DPPH Radical Scavenging Assay

The DPPH assay followed the Total Antioxidant Capacity Assay Kit. Test and standard solutions were mixed with the reagent, and after a 20-min dark reaction at room temperature, absorbance was measured at 515 nm using a microplate reader. Trolox served as the standard, and a series of Trolox standard solutions (0.18, 0.15, 0.12, 0.09, 0.06, 0.03 mM) were prepared. The total antioxidant capacity of each sample was expressed as µmol Trolox/g of herbal medicine dry weight [77].

#### 4.4.3. Ferric Reducing Ability of Plasma (FRAP) Assay

The FRAP assay followed the Total Antioxidant Capacity Assay Kit. The FRAP working solution was prepared by mixing TPTZ (2,4,6-tris(2-pyridyl)-s-triazine) diluent, TPTZ solution, and detection buffer solution at a ratio of 10:1:1 (*v*/*v*/*v*). After incubation at 37 °C and using within 1–2 h, test and standard solutions were mixed with the FRAP working solution in a 96-well plate and incubated at 37 °C for 3–5 min. After cooling to room temperature, the absorbance of the mixture was recorded at 593 nm using a microplate reader. FeSO_4_ served as the standard, and a series of FeSO_4_ standard solutions (0.15, 0.3, 0.6, 0.9, 1.2, 1.5 mM) were prepared. The total antioxidant capacity of each sample was expressed as µmol Fe(Ⅱ)/g of herbal medicine dry weight [78].

#### 4.4.4. Cupric Reducing Antioxidant Capacity (CUPRAC) Assay

The CUPRAC assay followed the Total Antioxidant Capacity Assay Kit (Cu^2+^ method). The Cu^2+^ working solution was prepared by mixing Cu^2+^ chelating agent solution and Cu^2+^ solution at a ratio of 50:1 (*v*/*v*). Test and standard solutions were mixed with the Cu^2+^ working solution in a 96-well plate, incubated at room temperature for 30 min, and absorbance was measured at 570 nm using a microplate reader. Trolox served as the standard, and a series of Trolox standard solutions (1, 0.5, 0.25, 0.125, 0.062, 0.031 mM) were prepared. The total antioxidant capacity of each sample was expressed as µmol Trolox/g of herbal medicine dry weight [79]. 

#### 4.4.5. Construction of Standard Curves

**Figure 2:**
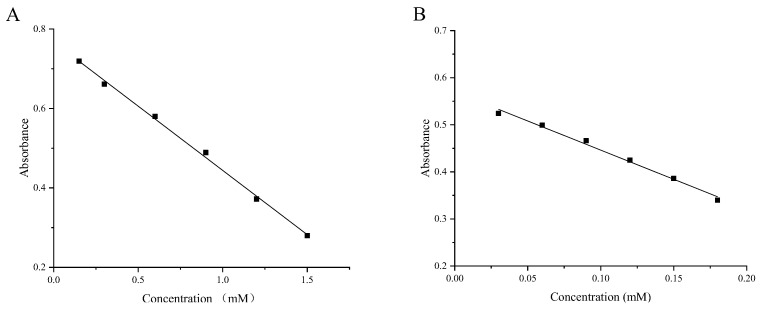

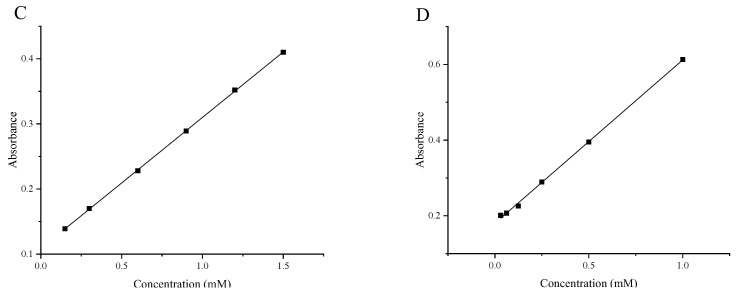
Standard curves for four antioxidant activity methods. ((**A**) Standard curve for Trolox in the ABTS method; (**B**) Standard curve for Trolox in the DPPH method; (**C**) Standard curve for Fe (Ⅱ) in the FRAP method; (**D**) Standard curve for Trolox in the CUPRAC method).

In this study, standard curves were constructed for the ABTS, DPPH, FRAP, and CUPRAC assays. The ABTS assay’s standard curve correlated absorbance values with known Trolox (6-hydroxy-2,5,7,8-etramethylchroman-2-carboxylic acid) concentrations, facilitating the calculation of Trolox Equivalent Antioxidant Capacity (TEAC) using a linear regression equation. Similarly, the DPPH assay utilized absorbance values correlated with various Trolox concentrations to generate a standard curve, enabling the conversion of experimental sample absorbance readings to TEAC values through a linear equation. For the FRAP assay, we created a standard curve by plotting absorbance against different concentrations of Fe(Ⅱ). The resulting linear regression equation was then applied to compute the FRAP of the tested samples. Likewise, the CUPRAC assay employed a standard curve established with known Trolox concentrations, utilizing the linear equation to quantify the CUPRAC of the samples. These standard curves not only formed the foundation for quantification and data analysis but also enhanced the accuracy and reliability of antioxidant activity assessments. Refer to Figure 2 for the standard curves, and Table 3 for the corresponding equations. The R^2^ values, ranging from 0.99133 to 0.99987, indicate excellent linearity, meeting the required standards.

#### 4.4.6. Statistical Analysis

The experiments were conducted in triplicate, and the results were reported as the mean ± standard deviation.

## 5. Conclusions

Through a comparative analysis of the non-volatile components and antioxidant activity in the branches and leaves of the traditional medicinal plant *L. cubeba*, we unveiled its diverse chemical composition, encompassing alkaloids, flavonoids, and organic acids across various categories. This underscores its potential as a valuable resource in traditional herbal medicine and the food industry. The distinct variations in alkaloids, organic acids, and flavonoids between the branches and leaves suggest potential differences in their suitability for various applications. Supporting our observations, UPLC-HRMS technology identified 72 constituents, unveiling significant chemical heterogeneity between the branches and leaves, with only eight components shared. This comprehensive characterization contributes to our understanding of *L. cubeba*’s chemical diversity. Regarding antioxidant activity, both the branches and leaves exhibited significant capabilities, with the branches demonstrating slightly higher efficacy. Consistent support from ABTS, DPPH, FRAP, and CUPRAC methods underscores the robust antioxidant potential within *L. cubeba*. This suggests *L. cubeba*’s promising role as a natural antioxidant with implications for health and therapeutic interventions. In summary, *L. cubeba* stands out as a botanical resource with unique chemical composition and potent antioxidant properties, providing theoretical support for its applications in medicine and the food industry. The identified chemical diversity and robust antioxidant activity lay a solid scientific foundation for further exploration. Future research endeavors could delve deeper into the biological activities and pharmacological effects of *L. cubeba* compounds, enhancing our understanding of its potential health benefits. This study contributes valuable insights for the ongoing exploration and utilization of *L. cubeba* in various fields.

## Figures and Tables

**Table 1:** Compounds identified in the methanol extract of *L. cubeba* branches and leaves by UPLC-HRMS.

No.	RT/min		Compounds	Molecular Formula	Error/ppm	*m*/*z*	Ion Mode	Compound Types	References
	Leaves	Branches							
1	0.851	–	2-Aminosuccinamic acid	C_4_H_8_N_2_O_3_	−1.54	131.04599	[M − H]^−^	alkaloids	[17]
2	0.88	–	Gluconic acid	C_6_H_12_O_7_	−1.22	195.05079	[M − H]^−^	carbohydrates	
3	0.89	–	L-Threonic acid	C_4_H_8_O_5_	−1.26	135.02972	[M − H]^−^	organic acids	[18]
4	0.893	–	D-(−)-Fructose	C_6_H_12_O_6_	−0.76	179.05597	[M − H]^−^	carbohydrates	[19]
5	0.904	–	D-(-)-Quinic acid	C_7_H_12_O_6_	−0.89	191.05595	[M − H]^−^	organic acids	[20]
6	0.908	–	L-Threonine	C_4_H_9_NO_3_	−0.3	120.06559	[M + H]^+^	amino acids	[21]
7	0.915	–	D-Glucosamine	C_6_H_13_NO_5_	0	180.0867	[M + H]^+^	carbohydrates	[22]
8	0.918	–	(2R)-2,3-Dihydroxypropanoic acid ^★^	C_3_H_6_O_4_	−1.37	105.01919	[M − H]^−^	organic acids	[23]
9	0.940	–	DL-Malic acid	C_4_H_6_O_5_	−1.53	133.01403	[M − H]^−^	organic acids	[24]
10	0.950	–	D-Serine ^★^	C_3_H_7_NO_3_	−0.06	106.04986	[M + H]^+^	amino acids	[25]
11	0.982	–	Proline	C_5_H_9_NO_2_	0.5	116.07066	[M + H]^+^	amino acids	[26]
12	0.998	–	Adenine	C_5_H_5_N_5_	0.91	136.06189	[M + H]^+^	alkaloids	[27]
13	1.019	–	Acetophenone	C_8_H_8_O	0.54	121.06486	[M + H]^+^	ketones	[28]
14	–	1.024	4-Methoxycinnamaldehyde	C_10_H_10_O_2_	−0.18	180.10186	[M + H]^+^	ethers	
15	–	1.140	Higenamine ^★^	C_16_H_17_NO_3_	0.31	272.1282	[M + H]^+^	alkaloids	[29]
16	1.219	–	Citric acid	C_6_H_8_O_7_	−0.32	191.01967	[M − H]^−^	organic acids	[30]
17	1.232	–	Nicotinic acid	C_6_H_5_NO_2_	0.14	124.03932	[M + NH_4_]^+^	alkaloids	[31]
18	1.238	–	Pipecolic acid	C_6_H_11_NO_2_	0.45	130.08631	[M + H]^+^	alkaloids	[32]
19	1.331	–	Methylmalonic acid	C_4_H_6_O_4_	−1.03	117.01921	[M − H]^−^	organic acids	[33]
20	1.335	–	Fumaric acid	C_4_H_4_O_4_	−1.05	115.00356	[M − H]^−^	organic acids	[34]
21	1.373	–	Isoleucine	C_6_H_13_NO_2_	0.35	132.10195	[M + H]^+^	amino acids	[35]
22	1.456	–	L-Norleucine	C_6_H_13_NO_2_	0.41	132.10196	[M + H]^+^	amino acids	[36]
23	–	1.615	L-N-Acetylphenylalaninol ^★^	C_11_H_15_NO_2_	−0.04	194.11755	[M + H]^+^	alkaloids	
24	–	1.989	(-)-Salsoline	C_11_H_15_NO_2_	−0.17	194.11752	[M + H]^+^	alkaloids	
25	2.089	–	Gentisic acid 5-O-β-glucoside ^★^	C_13_H_16_O_9_	−0.85	315.07189	[M − H]^−^	organic acids	
26	–	2.098	Indole-3-acetic acid	C_10_H_9_NO_2_	−0.03	176.07061	[M + H]^+^	alkaloids	[37]
27	–	3.396	trans-3-Indoleacrylic acid ^★^	C_11_H_9_NO_2_	−0.39	188.07054	[M + H]^+^	alkaloids	[38]
28	–	5.066	Norisoboldine	C_18_H_19_NO_4_	0.42	314.13882	[M + H]^+^	alkaloids	[39]
29	5.091	–	3-(4-Hydroxyphenyl)-3-oxopropyl β-D-glucopyranoside	C_15_H_20_O_8_	−0.82	327.10827	[M − H]^−^	phenols	[40]
30	5.888	5.261	Boldine	C_19_H_21_NO_4_	−0.07	328.15432	[M + H]^+^	alkaloids	[41]
31	5.486	–	Dihydrophaseic acid ^★^	C_15_H_22_O_5_	−0.41	281.13933	[M − H]^−^	organic acids	[42]
32	6.213	6.072	(S)-Boldine	C_19_H_21_NO_4_	0.26	328.15442	[M + H]^+^	alkaloids	[41]
33	6.636	6.487	Glaufinine	C_19_H_21_NO_4_	0.07	328.15436	[M + H]^+^	alkaloids	[43]
34	6.604	–	Kaempferol 3-sophoroside ^★^	C_27_H_30_O_16_	−0.28	609.14594	[M − H]^−^	flavonoids	[44]
35	–	6.765	Isocorydine	C_20_H_23_NO_4_	0.26	342.17007	[M + H]^+^	alkaloids	[45]
36	7.526	7.817	Rutin	C_27_H_30_O_16_	−0.69	609.14558	[M − H]^−^	flavonoids	[41]
37	–	7.117	Isoquercetin	C_21_H_20_O_12_	−0.28	463.08796	[M − H]^−^	flavonoids	[46]
38	7.316	–	Kaempferitrin	C_27_H_30_O_14_	−0.31	577.15588	[M − H]^−^	flavonoids	[47]
39	–	7.551	laurotetanine	C_19_H_21_NO_4_	0.07	328.15436	[M + H]^+^	alkaloids	[41]
40	7.557	–	5-(4-Hydroxypentyl)-1,3-benzenediol ^★^	C_11_H_16_O_3_	−0.11	197.1172	[M + H]^+^	phenols	
41	–	7.589	Corydine ^★^	C_20_H_23_NO_4_	−0.21	342.16991	[M + H]^+^	alkaloids	[43]
42	7.631	–	Trifolin ^★^	C_21_H_20_O_11_	−0.34	447.09298	[M − H]^−^	flavonoids	[48]
43	7.951	–	Cynaroside	C_21_H_20_O_11_	−0.26	447.09299	[M − H]^−^	flavonoids	[49]
44	–	8.048	Quercitrin	C_21_H_20_O_11_	−0.36	447.09312	[M − H]^−^	flavonoids	[41]
45	8.246	–	Cassythicin	C_19_H_19_NO_4_	0.33	326.13879	[M + H]^+^	alkaloids	[50]
46	–	8.349	Diosmin ^★^	C_28_H_32_O_15_	−0.77	607.16638	[M − H]^−^	flavonoids	[51]
47	–	8.503	Neohesperidin ^★^	C_28_H_34_O_15_	−0.78	609.18202	[M − H]^−^	flavonoids	[52]
48	–	8.644	Hesperetin	C_16_H_14_O_6_	0.57	303.08649	[M + H]^+^	flavonoids	[53]
49	–	8.648	Hesperidin	C_28_H_34_O_15_	0.65	611.19739	[M + H]^+^	flavonoids	[54]
50	9.196	–	Afzelin	C_21_H_20_O_10_	−0.63	431.0981	[M − H]^−^	flavonoids	[55]
51	9.443	–	Kaempferol ^★^	C_15_H_10_O_6_	0.05	287.05503	[M + H]^+^	flavonoids	[56]
52	–	9.930	N-p-Coumaroyltyramine	C_17_H_17_NO_3_	0.32	284.12821	[M + H]^+^	phenylpropanoids	[57]
53	–	10.391	Crebanine	C_20_H_21_NO_4_	0.3	340.15444	[M + H]^+^	alkaloids	[58]
54	10.576	10.454	Moupinamide	C_18_H_19_NO_4_	0.24	314.13876	[M + H]^+^	alkaloids	[59]
55	10.869	–	Tiliroside ^★^	C_30_H_26_O_13_	−0.92	593.12952	[M − H]^−^	flavonoids	[60]
56	14.447	14.029	15-Hexadecynoic acid ^★^	C_16_H_28_O_2_	−0.2	253.21616	[M + H]^+^	organic acids	
57	14.508	–	3-[[6-Deoxy-3,4-bis-O-[(2E)-3-(4-hydroxyphenyl)-1-oxo-2-propen-1-yl]-α-L-mannopyranosyl]oxy]-5,7-dihydroxy-2-(4-hydroxyphenyl)-4H-1-benzopyran-4-one	C_39_H_32_O_14_	−0.9	723.17128	[M − H]^−^	flavonoids	[61]
58	15.609	15.177	Lauryldimethylamine oxide ^★^	C_14_H_31_NO	−0.09	230.24782	[M + H]^+^	alkaloids	[62]
59	–	16.099	Aurantiamide acetate	C_27_H_28_N_2_O_4_	0.05	445.21222	[M + H]^+^	alkaloids	[63]
60	–	16.286	2-Amino-1,3,4-octadecanetriol ^★^	C_18_H_39_NO_3_	0.55	318.30045	[M + H]^+^	alcohols	[64]
61	–	16.290	4-Ethylbenzaldehyde	C_9_H_10_O	0.59	135.08052	[M + H]^+^	esters	[65]
62	–	16.297	4-Ethoxy ethylbenzoate	C_11_H_14_O_3_	−0.12	195.10155	[M + H]^+^	esters	[66]
63	16.529	–	Schinifoline ^★^	C_17_H_23_NO	−0.02	258.18524	[M + H]^+^	alkaloids	[67]
64	16.882	16.919	1,3:2,4-Di(p-ethylbenzylidene)sorbitol ^★^	C_24_H_30_O_6_	0.51	415.2117	[M + H]^+^	alcohols	
65	–	17.097	9-Oxo-ODE	C_18_H_30_O_3_	0.09	295.2268	[M + H]^+^	organic acids	[68]
66	–	17.368	13(S)-HOTrE	C_18_H_30_O_3_	0.08	293.21224	[M − H]^−^	organic acids	[69]
67	–	18.395	9-Oxo-10(E),12(E)-octadecadienoic acid	C_18_H_30_O_3_	0.4	295.22687	[M + H]^+^	organic acids	[70]
68	–	19.147	12-Oxo-10,15(Z)-phytodienoic acid ^★^	C_18_H_28_O_3_	0.45	293.21124	[M + H]^+^	organic acids	
69	–	19.440	Linoleoyl ethanolamide ^★^	C_20_H_37_NO_2_	0.28	324.28979	[M + H]^+^	alcohols	[71]
70	–	20.267	Sphingosine (d18:1) ^★^	C_18_H_37_NO_2_	0.6	300.28989	[M + H]^+^	alcohols	[72]
71	21.806	–	Muscone	C_16_H_30_O	−0.21	239.23689	[M + H]^+^	ketones	[73]
72	21.829	–	Pheophorbide A ^★^	C_35_H_36_N_4_O_5_	0.14	593.27594	[M + H]^+^	alkaloids	

–: not detected, ^★^: indicates compounds identified for the first time from *L. cubeba*.

**Table 2:** Antioxidant activity of methanol extracts from branches and leaves of *L. cubeba*.

Parts	ABTS (TEAC µmol/g)	DPPH (TEAC µmol/g)	FRAP (Fe(Ⅱ) µmol/g)	CUPRAC (TEAC µmol/g)
Branches	42.75 ± 3.94	7.55 ± 0.43	22.21 ± 0.53	42.65 ± 0.35
Leaves	57.78 ± 1.87	9.29 ± 0.39	28.68 ± 0.78	45.89 ± 0.27

**Table 3:** Standard curve equations for four antioxidant activity methods.

Method	Equation	R^2^
ABTS	y = −0.32338x + 0.76745	0.99755
DPPH	y = −1.23810x + 0.57000	0.99133
FRAP	y = 0.20124x + 0.10870	0.99987
CUPRAC	y = 0.43161x + 0.18027	0.99903

## Data Availability

Data are contained within the article and supplementary materials.

## Supplementary Materials

The following supporting information can be downloaded at: https://www.mdpi.com/article/10.3390/molecules29040788/s1, Figures S1–S4: UPLC-Q-Orbitrap HRMS chromatograms of *Litsea cubeba.*
