# Supplementary material for: Non-Volatile Component and Antioxidant Activity: A Comparative Analysis between Litsea cubeba Branches and Leaves
Source: Molecules. 2024 Feb 8;29(4):788. doi: 10.3390/molecules29040788 (PMC10892920; doi:10.3390/molecules29040788)
Supplement: Supplementary file 1 [file molecules-29-00788-s001.zip › molecules-2781745-supplementary.pdf]

Non-Volatile Component and Antioxidant Activity: A Comparative Analysis between *Litsea Cubeba* Branches and Leaves

Wei Dai [1], Boyi Li [2], Yanli Xiong [2], Liping Dai [1], Yuan Tian [3], Liangqian Zhang [3], Qi Wang [3,*] and Guoqiang Qian [2,*]

[1] Teaching and Experimental Center, Guangdong Pharmaceutical University, Guangzhou 510006, China; dai_gdpu_2018@gdpu.edu.cn (W.D.)

[2] School of Chinese Medicine, Guangdong Pharmaceutical University, Guangzhou 510006, China

[3] Key Laboratory of Xinjiang Phytomedicine Resource and Utilization, Ministry of Education, School of Pharmacy, Shihezi University, Shihezi 832003, China

* Correspondence: wq_pha@shzu.edu.cn (Q.W.); tgqqian@gdpu.edu.cn (G.Q.)

Figure S1. UPLC-Q-Orbitrap HRMS chromatograms of branches *Litsea cubeba* in negative ion mode
Figure S2. UPLC-Q-Orbitrap HRMS chromatograms of branches *Litsea cubeba* in positive ion mode
Figure S3. UPLC-Q-Orbitrap HRMS chromatograms of leaves *Litsea cubeba* in negative ion mode
Figure S4. UPLC-Q-Orbitrap HRMS chromatograms of leaves *Litsea cubeba* in positive ion mode

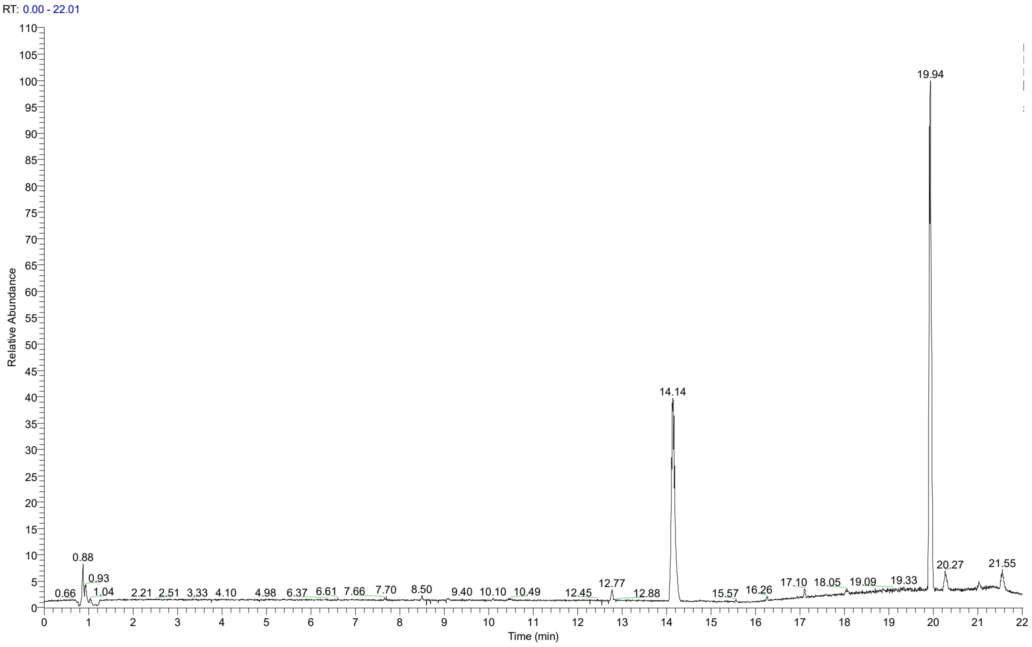

Figure S1. UPLC-Q-Orbitrap HRMS chromatograms of branches *Litsea cubeba* in negative ion mode

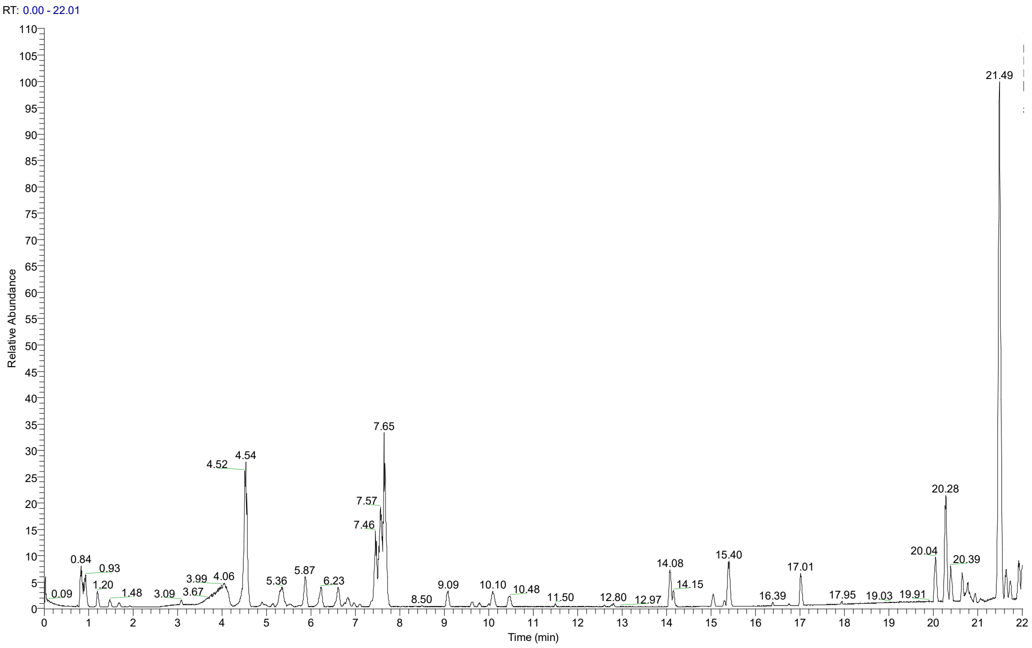

Figure S2. UPLC-Q-Orbitrap HRMS chromatograms of branches *Litsea cubeba* in positive ion mode

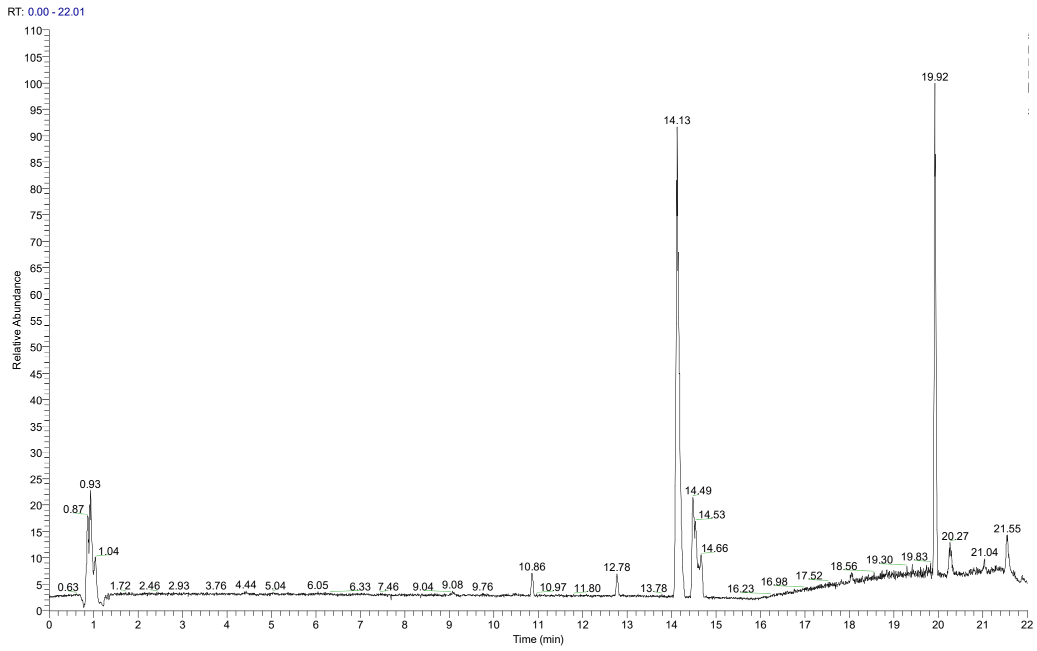

Figure S3. UPLC-Q-Orbitrap HRMS chromatograms of leaves *Litsea cubeba* in negative ion mode

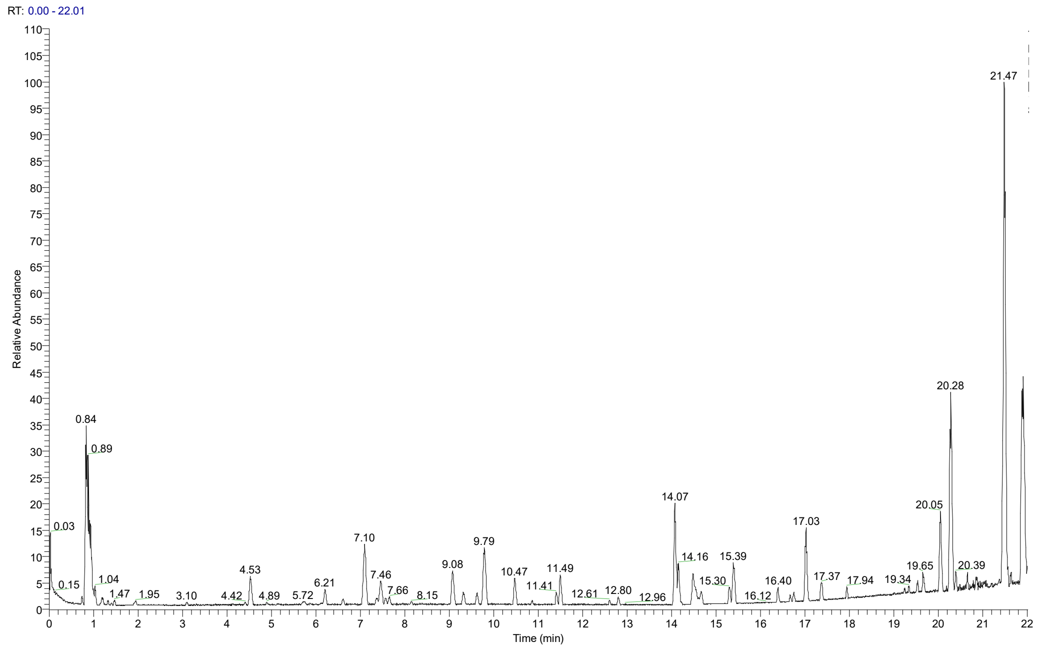

Figure S4. UPLC-Q-Orbitrap HRMS chromatograms of leaves *Litsea cubeba* in positive ion mode
